# A novel instrument for assessing health-related quality of life in French patients: translation and multi-centre validation of the Zurich chronic middle ear inventory (ZCMEI-21-Fr)

**DOI:** 10.1007/s00405-025-09447-0

**Published:** 2025-05-24

**Authors:** Thibault De Maesschalck, Raphaele Quatre, Mihaela Horoi, Alexandra Rodriguez, Pierre Sagnet, Christof Röösli, Alexander Huber, François Voruz, Pascal Senn, Sebastian Schmerber, David Bächinger

**Affiliations:** 1 Service of Otorhinolaryngology, Head and Neck Surgery, Department of clinical Neurosciences, University Hospital of Geneva, Geneva, Switzerland; 2 Center for Oto-Rhino-Laryngology - Maxillo-Facial and Head and Neck Surgery, Hôpital de la Tour, La Tour Medical Group, Meyrin, Switzerland; 3 Department of Oto-Rhino-Laryngology, Head and Neck Surgery, University Hospital, Grenoble, France; 4 Department of Otolaryngology-Head and Neck Surgery, CHU Saint-Pierre, Université Libre de Bruxelles, Brussels, Belgium; 5 Department of Otolaryngology, Head and Neck Surgery, CHL Luxembourg, Belair, Luxembourg; 6 Department of Otorhinolaryngology, Head and Neck Surgery, University Hospital Zurich, Zurich, Switzerland; 7 University of Zurich, Zurich, Switzerland

**Keywords:** Chronic otitis media, Health-related quality of life, ZCMEI-21, Translation, Validation

## Abstract

**Introduction:**

Chronic otitis media (COM) significantly impacts the quality of life of patients, yet existing assessment tools may not fully capture its multidimensional effects. The Zurich Chronic Middle Ear Inventory (ZCMEI-21) has emerged as a comprehensive and internationally recognized measure for evaluating health-related quality of life (HRQoL) in COM patients. However, a validated French version of the ZCMEI-21 is lacking, necessitating translation and validation to facilitate its use in French-speaking populations.

**Methods:**

A prospective multicentre study was conducted to translate and validate the ZCMEI-21 into French (ZCMEI-21-Fr). The translation process followed established guidelines, including professional translation, cognitive debriefing, and back-translation. The validation process involved administering the ZCMEI-21-Fr together with the generic EQ-5D questionnaire to French-speaking COM patients across multiple centers. Internal consistency, reliability, and validity were assessed using psychometric statistical methods.

**Results:**

A total of 102 French-speaking COM patients were included in the study. The ZCMEI-21-Fr demonstrated excellent internal consistency (Cronbach’s α = 0.89) and reliability across all subscales. Total ZCMEI-21-Fr scores correlated strongly with a question directly assessing HRQoL (*r* = 0.59, *p* < 0.0001). Moderate correlations were observed with the EQ-5D (*r* = 0.41, *p* < 0.0001).

**Discussion:**

The ZCMEI-21-Fr offers a valuable tool for assessing the psychosocial impact of COM in French-speaking patients. Its comprehensive coverage of HRQoL dimensions, including psychological effects, distinguishes it from existing instruments. The availability of a validated French version enhances the standardization of HRQoL assessment in COM patients on an international scale.

**Supplementary Information:**

The online version contains supplementary material available at 10.1007/s00405-025-09447-0.

## Introduction

Chronic otitis media (COM) is defined as a chronic tympanic membrane perforation with or without cholesteatoma. It affects approximately 1–4% of the population [1–3]. The most disabling symptoms are hearing deficit and a discharging ear, but other symptoms such as vertigo, tinnitus, or ear pressure and pain can also be present and have a profound effect on the health-related quality of life (HRQoL). The standard treatment is surgery with closure of the tympanic membrane perforation, with or without ossicular reconstruction, and eradication of cholesteatoma or diseased mucosa if present. Following surgery, hearing outcomes can be evaluated using audiometric tests, while recurrence of perforation can be assessed through clinical examination. However, these measures do not adequately capture the impact on the patient’s HRQoL. For instance, some patients may be more troubled by avoiding water in the ear post-surgery, while others might experience dissatisfaction with the quality of hearing.

Subjective outcomes in clinical studies are becoming increasingly important [4]. Treatment effectiveness is increasingly evaluated from the patient’s perspective. Usually, it is measured through generic health-related questionnaires. Since these instruments are not sensitive to capturing specific symptoms of COM, more precise, disease-specific questionnaires targeting COM have been developed recently. Most of them focus on the outcome of physical symptoms and hearing results. To date, only the COMQ-12 has been translated into French [5, 6]. However, it does not specifically address ear pain or vertigo. More importantly, the psychological impact is only covered by one question. Moreover, in the French validation study, the recall period was 3–6 months, which may introduce a significant recall bias [7]. The ZCMEI-21 was developed in 2016 [8]. It was originally written in German but has since been translated and validated in English, Italian, Japanese, Chinese and Turkish. It entails 21 questions, of which 7 concern the patient’s HRQoL, thus comprehensively covering the impact of COM on HRQoL [9–13]. Therefore, we translated the original version into French and validated it through a prospective multicentre study.

## Methods

### Ethical considerations

The study protocol was approved by each local Ethics Committee in accordance with the Declaration of Helsinki (2020 − 00490). Informed consent was obtained from all participants.

### Patients and study centres

Adult, French native patients with COM with or without cholesteatoma were included. The four participating centres were the otorhinolaryngology department of the university hospital of Geneva, Switzerland (HUG Genève), the university hospital of Grenoble, France (CHU Grenoble Alpes), the university hospital of Brussels, Belgium (CHU St-Pierre), and a regional hospital of Luxembourg city, Luxembourg (CHL Luxembourg). Data were collected from January 2021 to February 2023.

### Translation process

**Fig. 1:**
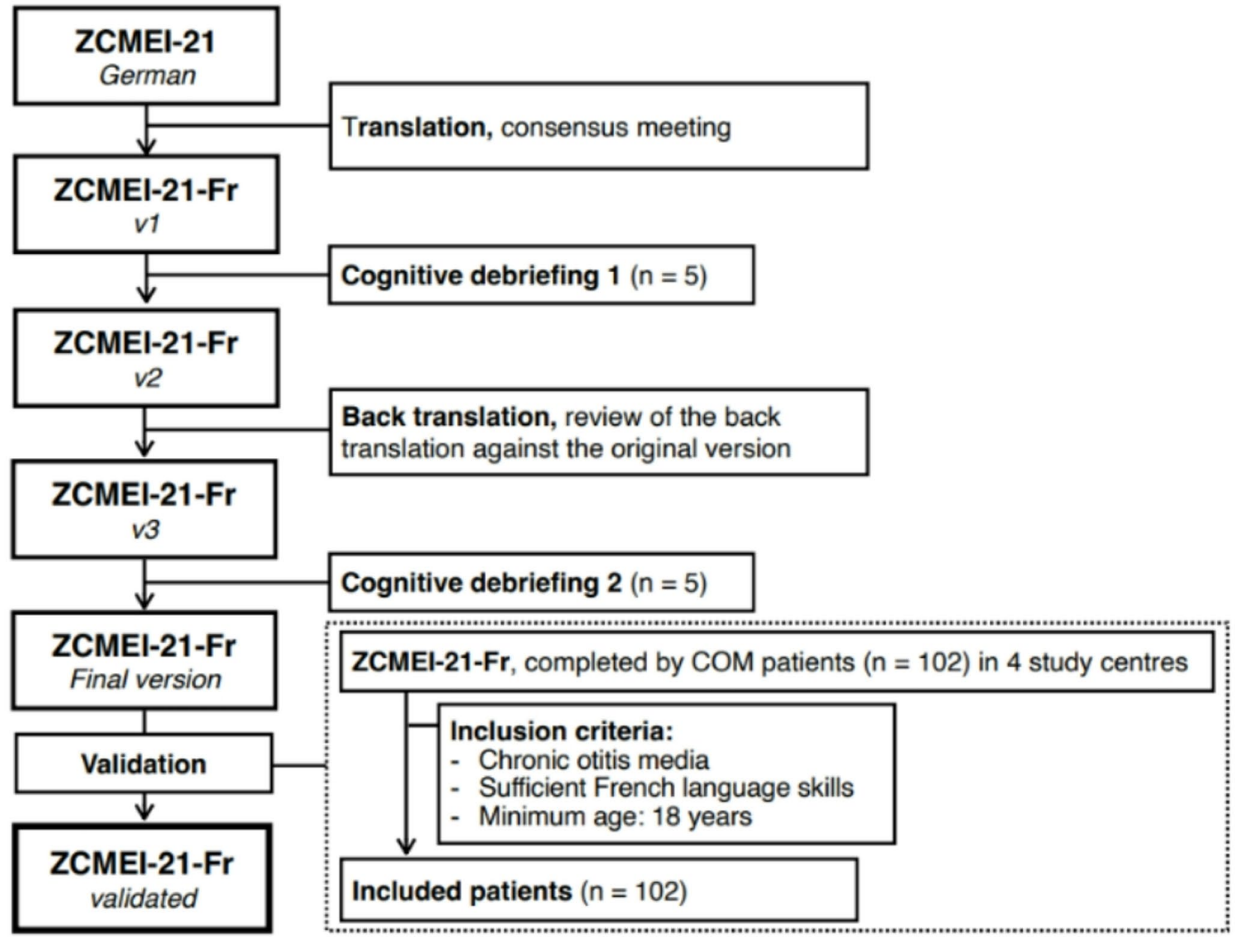
Flowchart illustrating study framework for translating and validating the French version of the ZCMEI-21 (ZCMEI-21-Fr)

A standardized approach for the translation and cultural adaptation of questionnaires according to the “Principles of Good Practice for the Translation and Cultural Adaptation Process for Patient-Reported Outcomes Measures” was used (Fig. 1). In the first phase, the translation of the original questionnaire was done by professional translators. This was then checked and corrected where needed by the principal investigator centre (Geneva). The questionnaire was tested with five patients, and difficulties in comprehension and interpretation were corrected. This version was then back-translated to German, compared with the original, and used to produce a final version after a second cognitive debriefing.

### Validation process

ZCMEI-21-Fr was administered to patients responding to the inclusion criteria at the end of the initial consultation. Participants were invited to complete two questionnaires related to their ear disorder. The total scores of the ZCMEI-21-Fr were computed by aggregating the individual item scores, which ranged from 0 to 4, resulting in a maximum possible score of 84. A higher total score on the ZCMEI-21-Fr indicates a more negative impact on HRQoL. Alongside the ZCMEI-21-Fr, we administered the five-level version of the EQ-5D questionnaire (EQ-5D-5 L, hereafter EQ-5D), which served as a generic HRQoL outcome measure due to its well-established utility for this purpose. The EQ-5D comprises five questions and a visual analogue scale (VAS), covering mobility, self-care, usual activities, pain/discomfort, and anxiety/depression. Responses to these questions generate an index value (descriptive system score) ranging from 0 to 1, with 1 representing perfect HRQoL. The VAS ranges from 0 to 100, with 100 indicating the best possible health state.

### Statistical analysis

Prior to data collection, all statistical tests were predetermined. The results are presented as means with standard deviations (SD) or as absolute numbers and percentages. To assess the correlation of each item with the overall score, the item-total correlation (ITC) was computed. To evaluate the reliability of the questionnaire, Cronbach’s α was utilized as an indicator of internal consistency. Frequency distribution was examined through histogram analysis and normality tests, including the D’Agostino and Pearson test and the Shapiro-Wilk test, where a p-value > 0.05 indicates a normal distribution. Criterion validity was determined by including an additional general question (question 22) directly addressing HRQoL. Concurrent validity was assessed by comparing the total scores of the ZCMEI-21-Fr and its sub-scores with the EQ-5D-5 L descriptive system and VAS scores using the Spearman’s rank correlation and linear regression analysis, including mean prediction intervals. Statistical analyses were performed using IBM SPSS Statistics for Windows, version 16.85 (IBM Corp., Armonk, NY, USA) and Prism (version 10.2.3 for Apple Macintosh, GraphPad Software). The significance level was set at *p* < 0.05.

## Results

A total of 102 patients were included (CHU Grenoble 46 patients of which 46 COM with cholesteatoma, HUG Geneva 43 patients of which 22 patients with COM with cholesteatoma, CHRU Brussels 10 and Luxembourg 3 patients). The mean age was 48 years, and there was a male predominance (65 males, 37 females). Most patients had COM with cholesteatoma (68%). The affected side distribution was rather symmetric with 45 right ears involved and 48 left ears. Nine had bilateral involvement. In total, 37% of the patients had a history of surgery. The complete demographics and patient characteristics are provided in Table 1.

**Table 1:** Demographic and clinical data of the patients within the cohort used for validating the ZCMEI-21-Fr

Males – no. (%)	65 (63.7%)
Females – no. (%)	37 (36.3%)
Age (SD)	47.7 (17.3)
COM, type– no. (%)	
COM without cholesteatoma	34 (33.3%)
COM with cholesteatoma	68 (66.7%)
Affected ear(s) – no. (%)	
Right	45 (44.1%)
Left	48 (47.1%)
Bilateral	9 (8.8%)
Previous surgery for COM– no. (%)	
Surgery (yes)	38 (37.3%)
Surgery (no)	64 (62.7%)
No. of Surgery (SD)	0.6 (1.1)

**Fig. 2:**
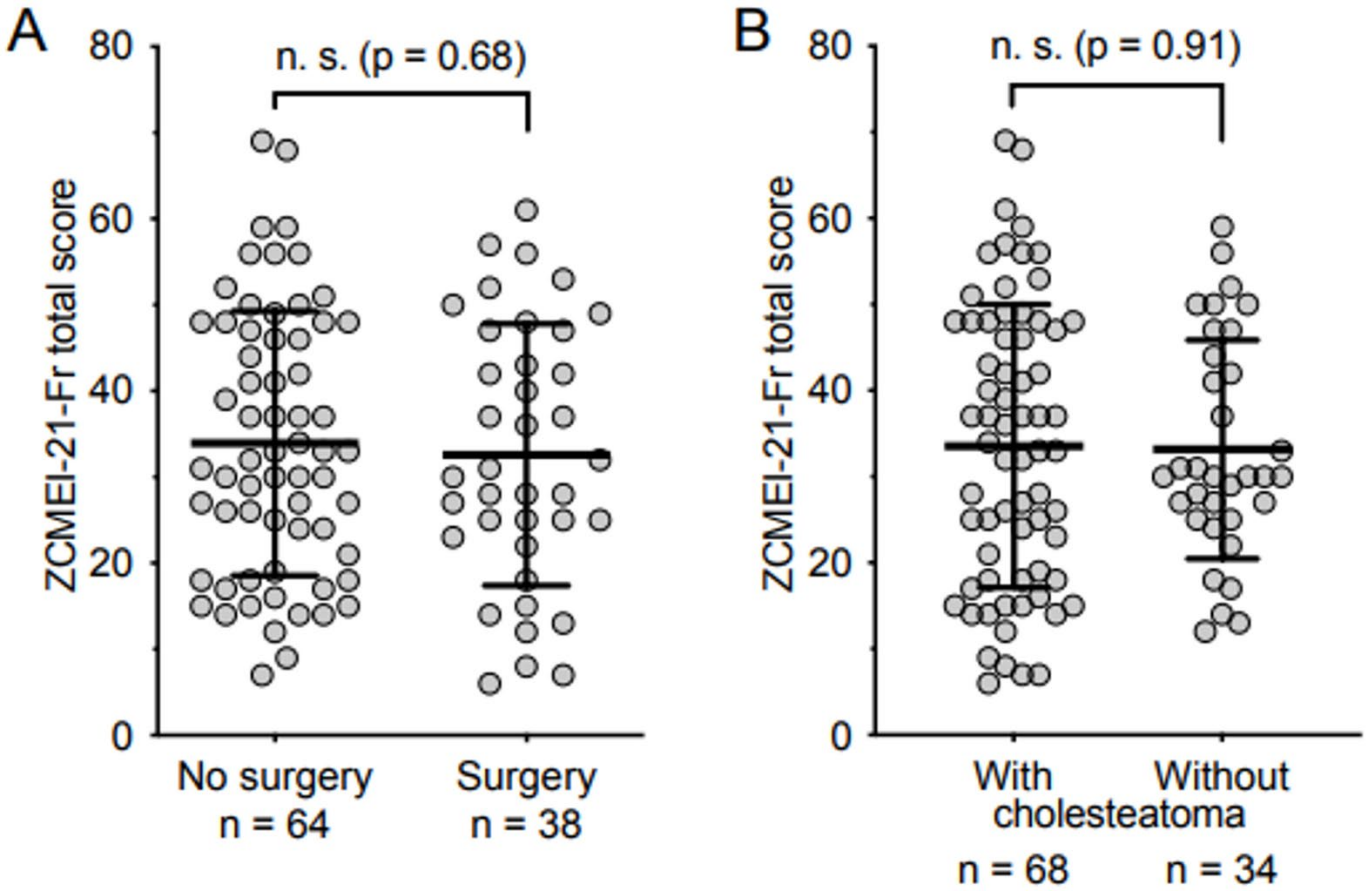
ZCMEI-21-Fr total scores in patients without and with history of surgery in (**A**) as well as with and without cholesteatoma (**B**)

No significant differences were observed in the total ZCMEI-21-Fr score between patients with or without a history of previous surgery and among groups with or without cholesteatoma (Fig. 2).The analysis revealed a Cronbach’s α of 0.89 for the entire questionnaire, indicating excellent internal consistency. Additionally, satisfactory to excellent internal consistency was observed for the individual subscales (subscale I, 0.78; subscale II, 0.77; subscale III, 0.85; subscale IV, 0.75).

**Fig. 3:**
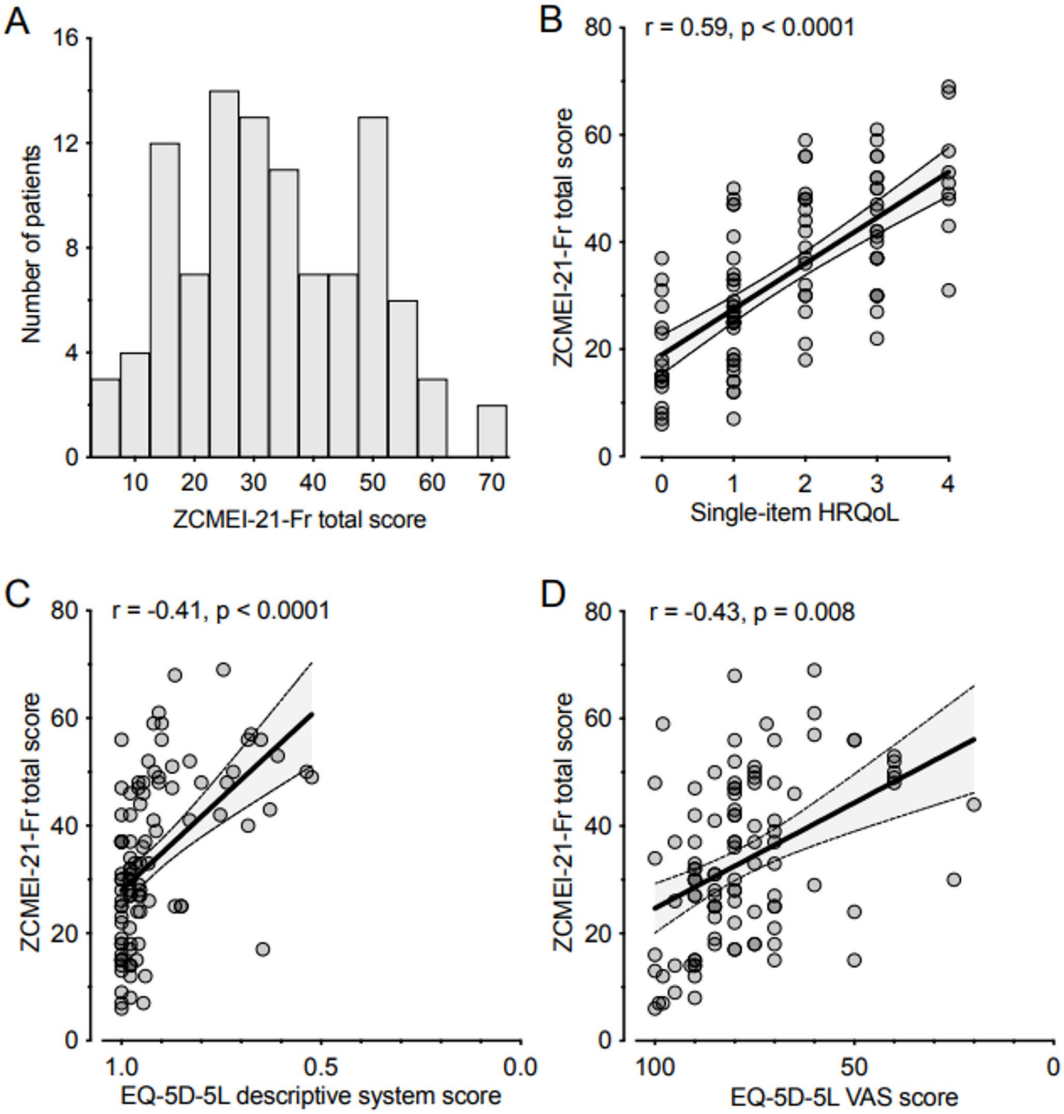
ZCMEI-21‐Fr total score frequency distribution and correlation to different HRQoL measures. **A** Best‐fitting Gaussian curve with bin width of 5 points on the x‐axis. **B** Spearman’s rank correlation for ZCMEI-21-Fr total scores and the added question #22, which directly assessed HRQoL (0 on the x-axis: no impact on HRQoL; 4 on the x-axis: huge impact on HRQoL; correlation). **C**–**D** Spearman’s rank correlation between ZCMEI‐21‐E total score and EQ‐5D descriptive systems score (**C**) as well the EQ‐5D VAS score (**D**)

Regarding single-item statistics, responses were well-distributed, encompassing the full range of answers for each question (Table 2). Item-total correlations for all items were consistently above 0.3, except for the final two questions related to the use of oral antibiotics and ear drops (Table 2). Furthermore, the distribution of total ZCMEI-21-Fr scores was examined. Both the inspection of the histogram (Fig. 3A) and the D’Agostino-Pearson normality test (*P* = 0.10) indicated a Gaussian distribution. A moderate correlation was identified between the ZCMEI-21-Fr total score and the question directly assessing HRQoL (added 22nd question; *r* = 0.59, *P* < 0.0001; Fig. 3B). Additionally, moderate correlations were observed between the ZCMEI-21-Fr total score and the EQ-5D descriptive system score (*r* = 0.41, *P* < 0.0001; Fig. 3C) and the EQ-5D VAS (*r* = 0.43, *P* < 0.0001; Fig. 3D). Lastly, correlations were found between the ZCMEI-21-Fr subscale scores and the EQ-5D descriptive system and VAS scores (Supplementary Fig. 1).

**Table 2:** Single item descriptive statistics of the ZCMEI-21-Fr. ITC item-total-correlation, min lowest value, max highest value

	mean	min-max	ITC
I. ear signs and symptoms			
1. ear pain	1.25	0–4	0.59
2. discharge	1.45	0–4	0.51
3. itching	1.54	0–4	0.42
4. feeling of pressure	1.35	0–4	0.49
5. balance	0.90	0–4	0.47
II. hearing			
6. tinnitus	2.12	0–4	0.49
7. hearing (filter question)	2.46	0–4	0.54
8. when many people speak at the same time	2.21	0–4	0.46
9. telephone, alarm-clock	1.10	0–4	0.34
10. fear of not hearing other people	2.20	0–4	0.57
III. psychosocial impact			
11. impact of ear symptoms on HRQoL	2.36	0–4	0.70
12. protection from water	1.17	0–4	0.65
13. activities with family and friends	1.39	0–4	0.53
14. in public (e.g. occupation, shopping)	0.80	0–4	0.56
15. making contact with other people	0.81	0–4	0.55
16. quality of sleep	1.49	0–4	0.66
17. sadness	2.12	0–4	0.56
18. fear that the ear problems may persist	1.79	0–4	0.41
IV. medical resources			
19. medical consultations	1.25	0–4	0.40
20. antibiotics (oral)	0.93	0–4	0.30
21. ear drops	1.04	0–4	0.18

## Discussion

The current study validates the French version of the ZCMEI-21 questionnaire (ZCMEI-21-Fr). The analysis of Cronbach’s α values for both the total and subscale scores demonstrated excellent internal consistency, thereby affirming the reliability of the ZCMEI-21-Fr. Regarding concurrent validity, we observed strong correlations between total ZCMEI-21-Fr scores and a direct measure of HRQoL in COM patients. Conversely, weak correlations were noted between ZCMEI-21-Fr total and subscale scores and the EQ-5D descriptive system score and VAS. Notably, the psychosocial impact questions displayed the strongest correlations, suggesting that the ZCMEI-21-Fr effectively captures the multifaceted nature of HRQoL. This underscores the notion that utilizing generic questionnaires may not adequately assess HRQoL in specific conditions such as COM. Multiple studies have shown that generic health-outcome questionnaires do not adequately assess or correlate with the HRQoL impact of COM. These questionnaires are limited in their ability to assess specific otologic conditions because they are either too general or focus only on a subset of COM-related symptoms, most commonly hearing loss [12].

Interestingly, comparable total ZCMEI-21‐Fr scores were found for patients with both COM with or without cholesteatoma regardless of whether they had surgery. This is in line with previous studies using the ZCMEI-21 [10–12]. These results suggest that HRQoL is impaired independent of the underlying type of COM. One could expect that patients who already had undergone surgery would suffer more of the disease. In addition, cholesteatoma patients are considered to have a more advanced form of chronic middle ear infection, but this does not come forward in our results. These results suggest that even patients with a more benign form of the disease suffer equally as much and once again reflect the importance of the quality-of-life aspects of the questionnaire as opposed to the questionnaires more oriented on physical symptoms.

Several disease-specific questionnaires have been developed for COM. The Chronic Ear Survey (CES), introduced in 2000, was the only validated COM-specific questionnaire until 2009 [14]. It assesses activity restriction, symptoms, and medical resource utilization but lacks coverage of psychological effects on HRQoL. This might explain discrepancies in studies correlating CES scores with disease severity [15–17]. Among the first disease-specific instruments to assess HRQoL in COM is the Chronic Otitis Media Outcome Test 15 (COMOT-15), which includes functional and subjective symptom assessments [18]. It has three subscales: Ear Symptoms, Hearing Function, and Mental Health, with added questions on QoL impact and outpatient visit frequency. While COMOT-15 emphasizes hearing impairment, it may inadequately address the individual influences of other symptoms like discharge, pain, vertigo, or tinnitus on QoL [17]. Another disease-specific instrument to assess HRQoL in COM is the COMQ-12 [5], which consists of 12 items grouped into four sections: Symptom severity lifestyle and work impact, health service impact, and general disease-related QOL item. This short questionnaire has been adapted and validated in different languages, including French [6, 19]. Although the COMQ-12 covers a broad spectrum of signs and symptoms, it is also heavily oriented toward the physical dimension. Of the 12 questions, seven addressed signs and symptoms. However, essential general components of HRQoL that are of importance in COM are not specifically covered, such as anxiety, depression, or social isolation. Therefore, to date, the ZCMEI-21-Fr questionnaire is the only one available in French that thoroughly evaluates the impact of the disease on the HRQoL [20]. As a side note, the ZCMEI-21 is the only questionnaire for which the minimal clinically important difference (MCID) is known, which indicates the smallest change in an outcome that patients perceive as meaningful [21]. Although the MCID of the original German version can serve as an estimate for other validated versions, future research will investigate the ZCMEI-21 MCID for other validated ZCMEI-21 versions.

## Conclusion

The ZCMEI-21-Fr has been successfully translated into French and validated through a prospective multicentre study. It is particularly effective in assessing the psychosocial impact of COM, alongside with physical impact, making it a valuable tool for both clinical practice and research. The questionnaire is applicable to patients with or without cholesteatoma and is independent of surgical history, ensuring broad utility across different patient populations. Additionally, incorporating a French version of the ZCMEI-21 into the existing array of validated ZCMEI-21 versions in multiple languages facilitates the standardization of HRQoL assessment of COM on a global scale.

## Electronic supplementary material

Below is the link to the electronic supplementary material.

Supplementary material 1
