# Supplementary material for: A novel instrument for assessing health-related quality of life in French patients: translation and multi-centre validation of the Zurich chronic middle ear inventory (ZCMEI-21-Fr)
Source: Eur Arch Otorhinolaryngol. 2025 May 24;282(10):5123–9. doi: 10.1007/s00405-025-09447-0 (PMC12518408; doi:10.1007/s00405-025-09447-0)

**Supplementary Fig. 1.** ZCMEI‐21‐Fr subscale scores correlated to the EQ‐5D descriptive system and VAS scores. Subscale I (ear signs and symptoms; A–B), subscale II (hearing; C–D), and subscale III (psychosocial impact; E–F) weakly correlated to the EQ‐5D descriptive system scores as well as to the EQ‐5D VAS scores, whereas all correlation coefficients were statistically significant (P < 0.0001 for all correlation coefficients).


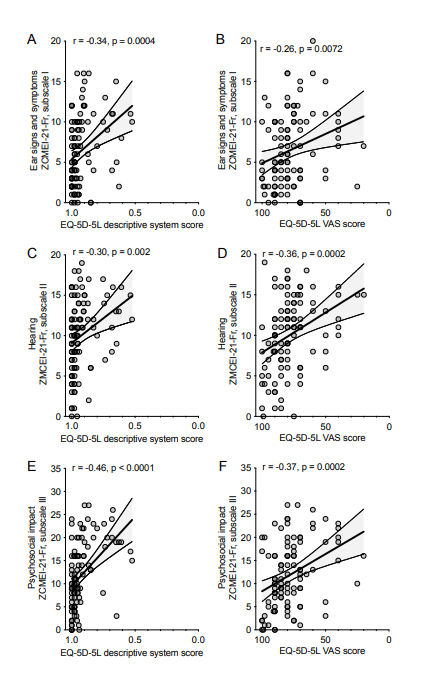

Supplement: Supplementary file 1 — Supplementary material 1 [file 405_2025_9447_MOESM1_ESM.docx]
